# Monitoring diffuse volcanic degassing during volcanic unrests: the case of Campi Flegrei (Italy)

**DOI:** 10.1038/s41598-017-06941-2

**Published:** 2017-07-28

**Authors:** C. Cardellini, G. Chiodini, F. Frondini, R. Avino, E. Bagnato, S. Caliro, M. Lelli, A. Rosiello

**Affiliations:** 1 Dipartimento di Fisica e Geologia, Università degli Studi di Perugia, via Pascoli snc, 06123 Perugia, Italy; 2 Istituto Nazionale di Geofisica e Vulcanologia, Sezione di Bologna, via D. Creti 12, 40128 Bologna, Italy; 3 Istituto Nazionale di Geofisica e Vulcanologia, Sezione di Napoli Osservatorio Vesuviano, via Diocleziano 328, 80124 Napoli, Italy; 4 Consiglio Nazionale delle Ricerche, Istituto di Geoscienze e Georisorse, Via G. Moruzzi 1, 56124 Pisa, Italy

## Abstract

In volcanoes with active hydrothermal systems, diffuse CO_2_ degassing may constitute the primary mode of volcanic degassing. The monitoring of CO_2_ emissions can provide important clues in understanding the evolution of volcanic activity especially at calderas where the interpretation of unrest signals is often complex. Here, we report eighteen years of CO_2_ fluxes from the soil at Solfatara of Pozzuoli, located in the restless Campi Flegrei caldera. The entire dataset, one of the largest of diffuse CO_2_ degassing ever produced, is made available for the scientific community. We show that, from 2003 to 2016, the area releasing deep-sourced CO_2_ tripled its extent. This expansion was accompanied by an increase of the background CO_2_ flux, over most of the surveyed area (1.4 km^2^), with increased contributions from non-biogenic source. Concurrently, the amount of diffusively released CO_2_ increased up to values typical of persistently degassing active volcanoes (up to 3000 t d^−1^). These variations are consistent with the increase in the flux of magmatic fluids injected into the hydrothermal system, which cause pressure increase and, in turn, condensation within the vapor plume feeding the Solfatara emission.

## Introduction

Volcanoes emit volatiles through active plumes, fumarolic vents and zones of diffuse soil degassing^1, 2^. Emitted volatiles may represent the surface manifestation of magma degassing^2–6^ providing useful information for the better understanding of processes occurring at depth, for assessing the state of activity of a volcano and, potentially, for forecasting the likelihood of a volcano erupting. Because of the relatively low solubility of CO_2_ in silicate melt^7–9^, CO_2_ is particularly useful as it exsolves from magma at greater depths than other volatile species, and therefore can reflect deep processes^10–13^. Diffuse CO_2_ degassing may represent the dominant mode of volcano degassing at calderas and volcanoes with hydrothermal activity (see for example refs 14–19), Several calderas have shown signs of unrest (ref. 20 and refs therein), however in some cases is problematic to understand if these are driven by magmatic activity (e.g., magma intrusion) or are related to hydrothermal dynamics (e.g., pressurization of the hydrothermal system)^3, 12, 21–23^.

**Figure 1:**
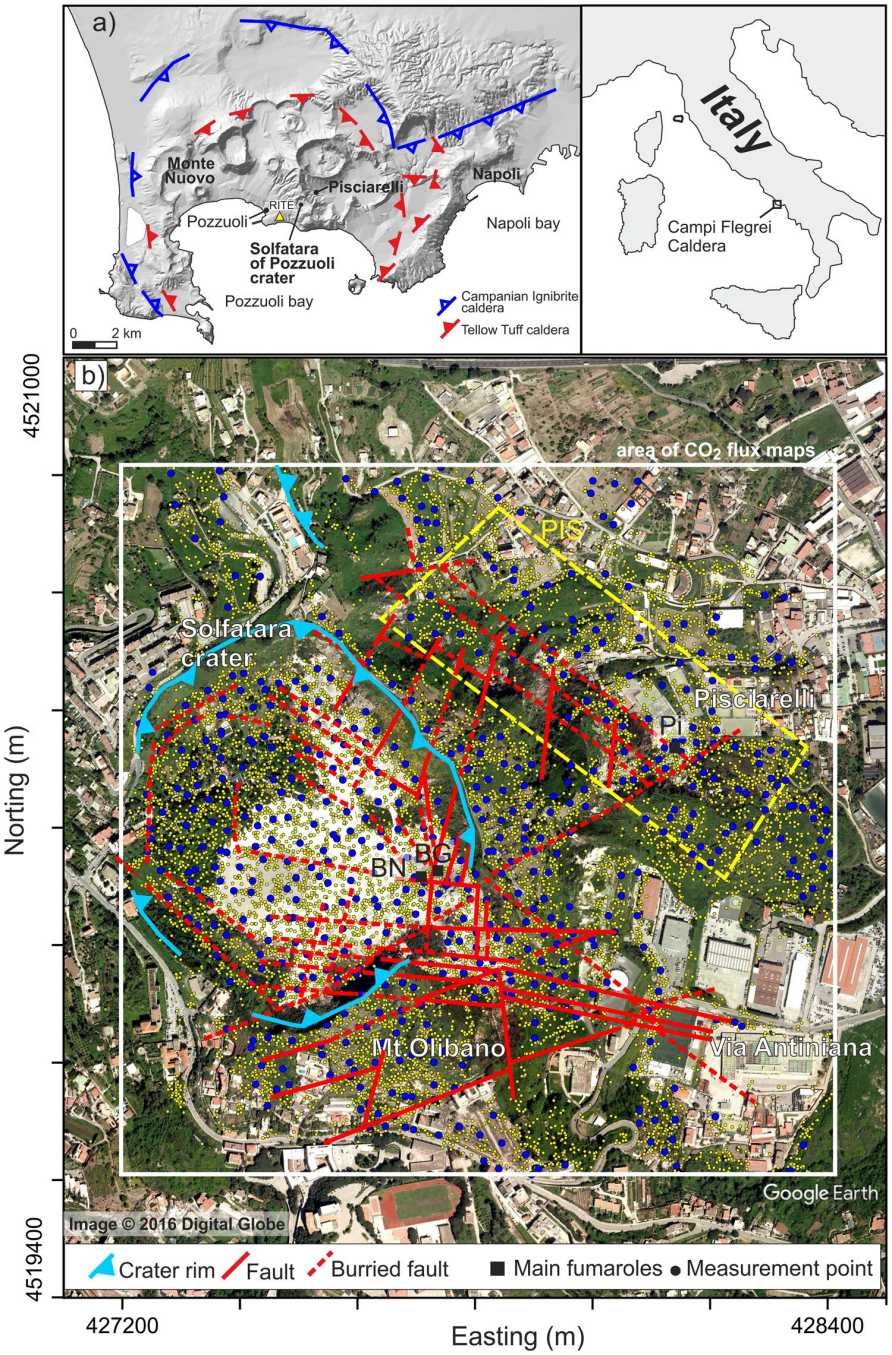
(**a**) Location of Solfatara of Pozzuoli and Campi Flegrei caldera; both maps were obtained using the open-access digital elevation model of Italy, TINITALY/01^75^. (**b**) Map of surveyed area. In the map are reported: the location of all the CO_2_ flux measurements (yellow dots) and, as example, the locations of CO_2_ measurements of January 2016 survey (blue dots); the location of Bocca Nuova (BN), Bocca Grande (BG) and Pisciarelli (Pi) fumaroles; the main tectonic structures^60^; the area considered for the mapping of CO_2_ fluxes (white box); the area considered for the computation of the total CO_2_ output from Pisciarelli area (PIS, box indicated by the dashed yellow line). Coordinates are reported as meters projection UTM European Datum 50. All the maps were realized with the software Surfer, Version 11.0.642 (http://www.goldensoftware.com/products/surfer).

Diffuse degassing is the main way in which CO_2_ is emitted by Solfatara di Pozzuoli15 (Solfatara hereafter), located in the centre of the restless Campi Flegrei caldera^24, 25^ (CFc, Fig. 1).

A reliable technique for measurement of soil diffuse CO_2_ degassing (accumulation chamber, AC, see Methods) was developed at the end of 20^th^ century, rapidly becoming extensively used in volcanological sciences^26, 27^. Solfatara is one of the first sites in the world where this technique, together with those used in soil CO_2_ diffuse degassing data analysis, were tested and improved throughout the 1990s^28, 29^. In general, Solfatara has become a natural laboratory for testing new types of measurements for the gas flux from hydrothermal sites based on the *in situ* and remote sensing determination of CO_2_^12, 30–33^.

Hydrothermal activity at Solfatara results in numerous fumaroles and in widespread hot soils and diffuse gas emissions. The thermal energy released by diffuse degassing at Solfatara is by far the main mode of energy release from the entire Campi Flegrei caldera^15^.

**Figure 2:**
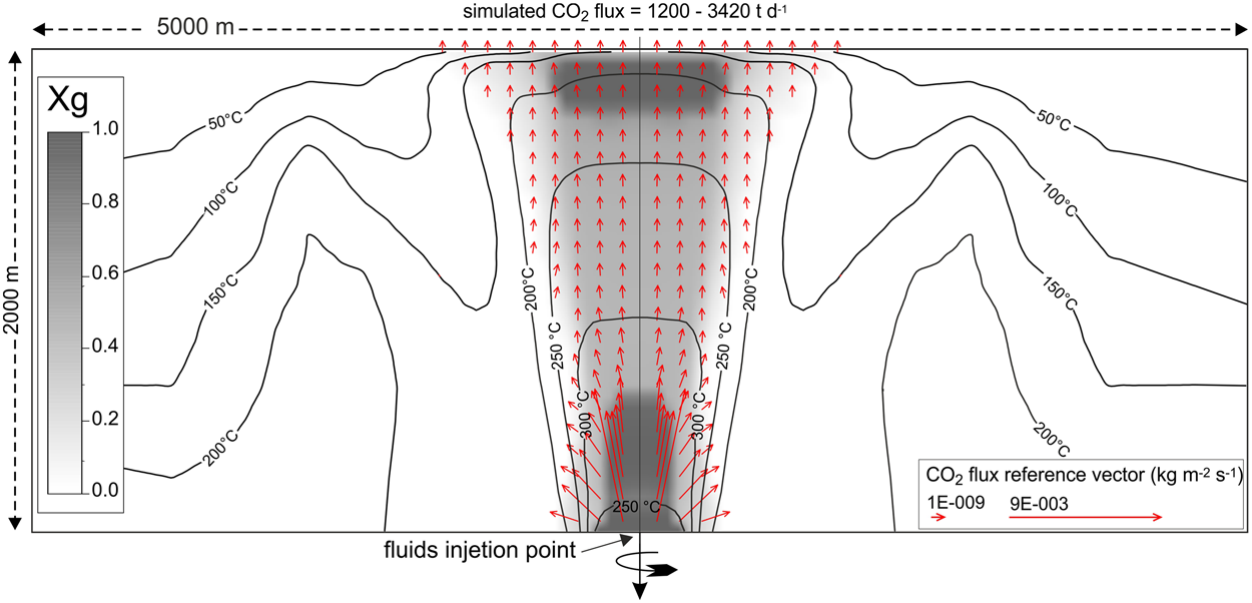
Computational domain of TOUGH2 simulations. The temperature, the volumetric gas fraction Xg (different shades of grey) and the CO_2_ flux vectors refer to initial steady-state conditions. Details of the modelling are reported in ref. 3.

The diffuse degassing at Solfatara is fed by a 1.5–2 km-deep subterranean vapor plume, the presence of which was first hypothesised based on geochemical conceptual models of the fumaroles^15, 34–40^ and subsequently highlighted by the re-interpretation of seismic tomography of CFc^25, 41, 42^. The same concept, i.e. the presence of a subterranean vapor plume, is returned by TOUGH2 modelling of the hydrothermal system feeding the Solfatara fumarolic field^3, 4^ (Fig. 2).

The vapor plume connects the surface to a hydrothermal zone at about 2 km depth^15, 25, 37, 39, 43, 44^, where the meteoric fluids mix with magmatic gases coming from a deeper zone at about 3–6 km depth^45–47^.

The emitted CO_2_ is thought to derive mainly from magma degassing^34^, even if we cannot exclude a minor contribution from decarbonation of hydrothermal calcite^48^. A relatively positive (−1.3‰ ± 0.4‰ ref. 48) carbon isotope signature of the fumarolic CO_2_, as well of the CO_2_ involved in the past deposition of hydrothermal calcites^48^ indicates a primary origin of the CO_2_ from a mantle metasomatised by crustal fluids^34, 49, 50^.

In this work, the results of 30 diffuse CO_2_ flux surveys performed at Solfatara from 1998 to 2016 are presented and discussed. The CO_2_ soil fluxes were measured over an area of ~1.2 × 1.2 km, including the Solfatara crater and the hydrothermal site of Pisciarelli (Fig. 1), using the AC (see Method). Each survey consisted of a number of CO_2_ flux measurements varying from 372 to 583 (Table 1) resulting in a total of 13,158 measurements.

**Table 1:** Summary statistics of the CO_2_ flux dataset, statistical parameters of the partitioned CO_2_ flux populations, areal extents of the Solfatara DDS and total CO_2_ output estimates.

Date	Meas. n.	CO_2_ flux range	CO_2_ flux mean	CO_2_ HF population		CO_2_ LF population		DDS extent	Total CO_2_ output
		(g m^−2^ d^−1^)	(g m^−2^ d^−1^)	Fraction (%)	Mean flux (g m^−2^ d^−1^)	Fraction (%)	Mean flux (g m^−2^ d^−1^)	(m^2^)	(t d^−1^)
01/12/1998	402	1.9–51940	1268 ± 4118	39	3730 ± 980	61	19.4 ± 1.26	454585	1329 ± 122
01/07/2000	414	3.0–30987	1300 ± 3791	39	4590 ± 1351	61	23.9 ± 1.50	455188	1513 ± 146
18/02/2003	398	0.8–51978	842 ± 3301	59	2248 ± 1116	41	14.4 ± 1.17	545655	784 ± 85
01/07/2003	391	3.8–12823	647 ± 1604	60	1274 ± 283	40	32.7 ± 2.41	843293	745 ± 61
09/04/2004	413	2.7–40123	1260 ± 4004	59	2778 ± 775	41	46.5 ± 2.38	990716	1351 ± 164
30/08/2004	404	1.8–33116	863 ± 2708	65	1421 ± 323	35	46.5 ± 3.29	961003	889 ± 96
24/03/2005	423	1.6–18515	692 ± 2708	57	1397 ± 273	43	42.5 ± 2.71	890046	941 ± 89
17/10/2005	408	3.9–24151	1008 ± 2559	37	2814 ± 452	63	88.0 ± 8.19	914215	1054 ± 103
27/05/2006	403	0.5–34560	716 ± 2583	40	1559 ± 217	60	65.6 ± 4.51	978181	1155 ± 147
30/10/2006	400	3.5–39548	1194 ± 3415	47	2629 ± 566	53	74.7 ± 6.34	917084	1242 ± 135
01/03/2007	372	8.4–28834	1140 ± 3339	54	2254 ± 544	46	46.9 ± 2.58	923259	1202 ± 120
27/06/2008	427	6.6–14981	804 ± 1910	56	1550 ± 277	44	57.6 ± 3.40	1058003	1099 ± 107
18/03/2009	473	1.8–28317	1216 ± 3257	50	3970 ± 1297	50	40.8 ± 2.49	823329	1501 ± 171
07/07/2009	503	1.1–36798	1131 ± 3311	58	1952 ± 342	42	42.4 ± 3.01	949466	1201 ± 107
24/11/2009	451	1.5–39245	1351 ± 3975	48	3041 ± 697	52	56.7 ± 5.33	870470	1271 ± 150
17/05/2010	505	3.4–35462	875 ± 2830	51	1885 ± 442	49	41.5 ± 2.71	788067	888 ± 97
10/03/2011	424	0.7–25682	985 ± 2789	43	2603 ± 512	57	41.3 ± 2.87	677936	1027 ± 100
18/06/2012	470	2.2–27017	1289 ± 3127	52	2831 ± 458	48	83.8 ± 6.93	1139904	1325 ± 135
01/10/2012	396	6.8–16325	1312 ± 2736	48	3709 ± 773	52	98.7 ± 7.52	1229796	1524 ± 164
28/02/2013	438	1.3–46648	1462 ± 3879	41	3917 ± 683	59	85.8 ± 7.40	1029707	1523 ± 151
20/05/2013	463	1.3–55829	1167 ± 3784	53	2266 ± 398	47	50.6 ± 2.69	1095011	1404 ± 170
02/07/2013	416	2.8–25683	1110 ± 2649	50	2791 ± 580	50	74.6 ± 4.79	1130882	1162 ± 125
25/09/2013	422	0.5–20398	1076 ± 2554	57	2316 ± 455	43	56.1 ± 4.13	1090043	1116 ± 138
06/06/2014	468	10.5–27066	1224 ± 2569	60	2359 ± 363	40	63.2 ± 3.29	1113823	1113 ± 149
22/09/2014	398	3.1–25774	1565 ± 3524	40	4450 ± 704	60	121 ± 12.2	1151089	1530 ± 169
26/01/2015	397	1.5–32197	2557 ± 5139	46	6579 ± 964	54	117 ± 13.8	1168794	2815 ± 318
20/03/2015	434	6.9–29509	1863 ± 4131	40	5478 ± 825	60	76.6 ± 5.45	996530	1922 ± 210
06/11/2015	508	1.8–41479	1704 ± 4438	49	3846 ± 684	51	65.4 ± 4.33	919573	1343 ± 153
20/01/2016	583	1.1–57240	1847 ± 4917	46	5538 ± 1156	54	70.4 ± 6.54	869627	1750 ± 221
06/06/2016	554	0.4–72434	2001 ± 5587	40	5235 ± 801	60	136 ± 10.3	1176213	1563 ± 165

The measured CO_2_ flux mean are presented as mean ± SD. The mean CO_2_ flux of statistically partitioned population are presented as mean ± SD of the mean. The total CO_2_ output is presented as mean ± SD.

This data set, entirely reported in Supplementary Information Dataset S1, is one of the largest datasets made anywhere^18, 51, 52^ on a single degassing volcanic-hydrothermal system. It is particularly relevant in the framework of volcanological sciences because it was acquired during a long period of unrest at CFc. Aside from making this large data set available, our main aim is to investigate how CO_2_ emissions varied during the progress of the CFc volcanic unrest that is characterised by accelerating geophysical and geochemical signals^4, 25, 53, 54^. Since the 1950’s, CFc underwent several episodes of ground uplift and deformation^55^, generally accompanied by seismic swarms and abruptly followed by significant changes in the composition of fumaroles^4^. The largest bradyseismic episode occurred from 1982 to 1984 with a total uplift of 1.79 m. After about twenty years of prevailing subsidence, a new unrest phase started at the beginning of the new millennium. This crisis, still ongoing, deviates from the previous episodes due to the long duration and the clear acceleration of signals, which were recently interpreted as being mainly caused by an ongoing heating of the system^3, 25^, and, regarding the 2012–2013 accelerated ground uplift, by the intrusion of magma at shallow depths^53, 56^.

In the following discussion, measured CO_2_ fluxes will be compared with the results of a recently published model^3^ that simulated the effects of the injection of magmatic fluids into a virtual hydrothermal system, ideally representing the system feeding CO_2_ emissions at Solfatara. With respect to previous physical models of the system, the injected magmatic fluids in ref. 3 are progressively richer in water, thus explaining the heating of the system. Here, for the first time, the modelled CO_2_ fluxes are compared with those observed during the ongoing crisis at CFc.

Finally, a further objective of the work is the comparison of the long series of CO_2_ flux data from Solfatara, a hydrothermally active volcano, with both measured geothermal systems, and CO_2_ fluxes from active volcanic plumes on which global volcanic CO_2_ emissions are largely based.

## Results

### Statistical distribution of CO_2_ flux

**Figure 3:**
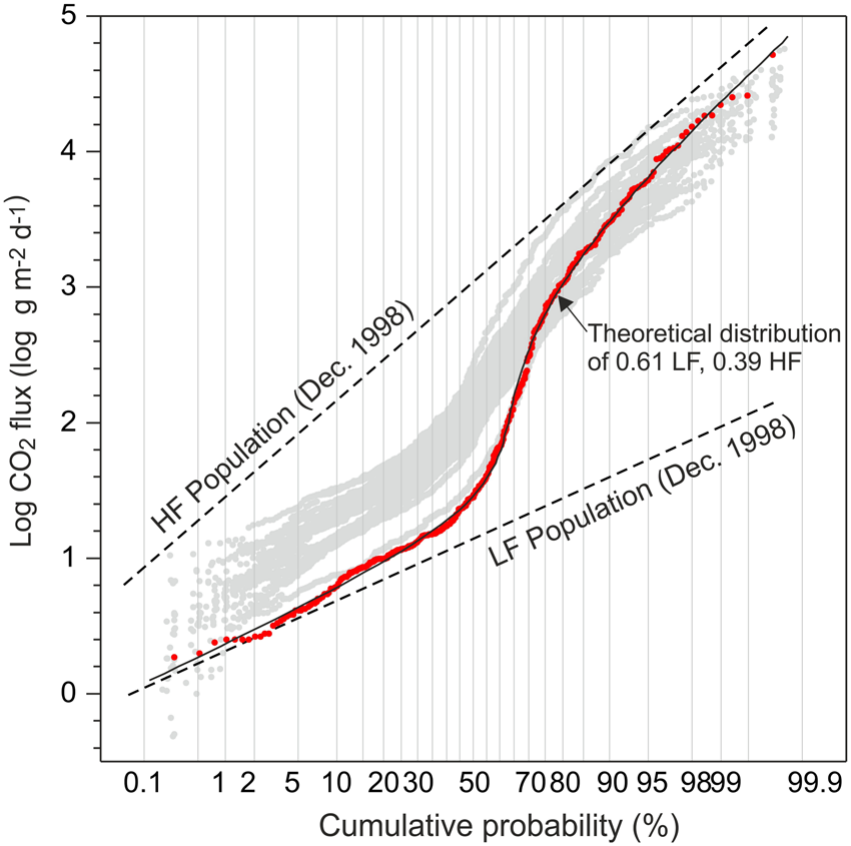
Log probability plot of the soil CO_2_ fluxes. Grey dots show log CO_2_ flux data statistical distribution of all the surveys. As example are reported, the December 1998 dataset with red dots, the partitioned population (HF and LF populations) for this dataset using Sinclair method^71^ (see Methods) with the dashed lines, and the theoretical distribution resulting from the combination of the HF and LF populations in the proportion 61% LF and 39% HF with the solid line.

The measured CO_2_ flux of each survey is distributed in a wide range of values from >0.4 g m^−2^ d^−1^ up to 72,000 g m^−2^ d^−1^ (Table 1). The logarithmic probability plot of Fig. 3 reveals that the CO_2_ fluxes of each survey plot as a curve with an inflection point. Such curves correspond to a bimodal statistical distribution, resulting from the combination of two CO_2_ flux log-normal populations (see Methods), which could indicate the occurrence of two CO_2_ flux sources^29, 57^, different mechanisms of gas transport, different permeability of the soil, etc.

The statistical distribution of CO_2_ flux of each survey was modelled (see Methods) with the combination of a population characterised by a high mean CO_2_ flux value, HF population, and one characterised by a lower mean CO_2_ flux value, LF population (Fig. 3).

The mean CO_2_ flux value of LF and HF populations range from 14 g m^−2^ d^−1^ to 135 g m^−2^ d^−1^ and from 1,270 g m^−2^ d^−1^ to 6,580 g m^−2^ d^−1^ respectively (Table 1). The high mean CO_2_ flux values of HF populations clearly indicate that they are fed by the CO_2_ up-rising from the underlying hydrothermal system (see for example refs 15, 28, 29, 57 and 58). The mean flux value of LF populations varies within a range that precludes the possibility that they have a purely biologic source for the entire period. In fact, the mean biogenic CO_2_ flux from a wide variety of ecosystems ranges from 0.2 g m^−2^ d^−1^ to 21 g m^−2^ d^−1^ (ref. 57 and reference therein). Roughly in agreement with these typical biogenic-derived fluxes, the coupled analyses of the CO_2_ flux and the isotopic composition of the CO_2_ efflux performed at Solfatara in March 2007 (see Supplementary Information Fig. S1) indicated a mean biogenic CO_2_ flux of 26 (±3) g m^−2^ d^−1^ (ref. 58). Since 2004, the mean CO_2_ flux of LF populations increased to 136 g m^−2^ d^−1^ (Table 1), i.e. values much higher than those associated with a purely biological source. The seasonal variability of the biologic production of CO_2_ within the soil, resulting in soil CO_2_ flux lower in autumn-winter and higher in spring-summer seasons (see for example ref. 59), cannot account for the temporal variation of the mean CO_2_ flux of LF population (Table 1). Therefore, even though mean CO_2_ flux values of LF populations are consistently 1 to 2 orders of magnitude lower than mean CO_2_ flux values of HF populations, since 2004, the LF population clearly represents a mixture of biogenic and deeply-derived CO_2_. This suggests that deep-sourced CO_2_ degassing widely affects both Solfatara crater and its surroundings.

### Mapping of diffuse degassing and total CO_2_ output

**Figure 4:**
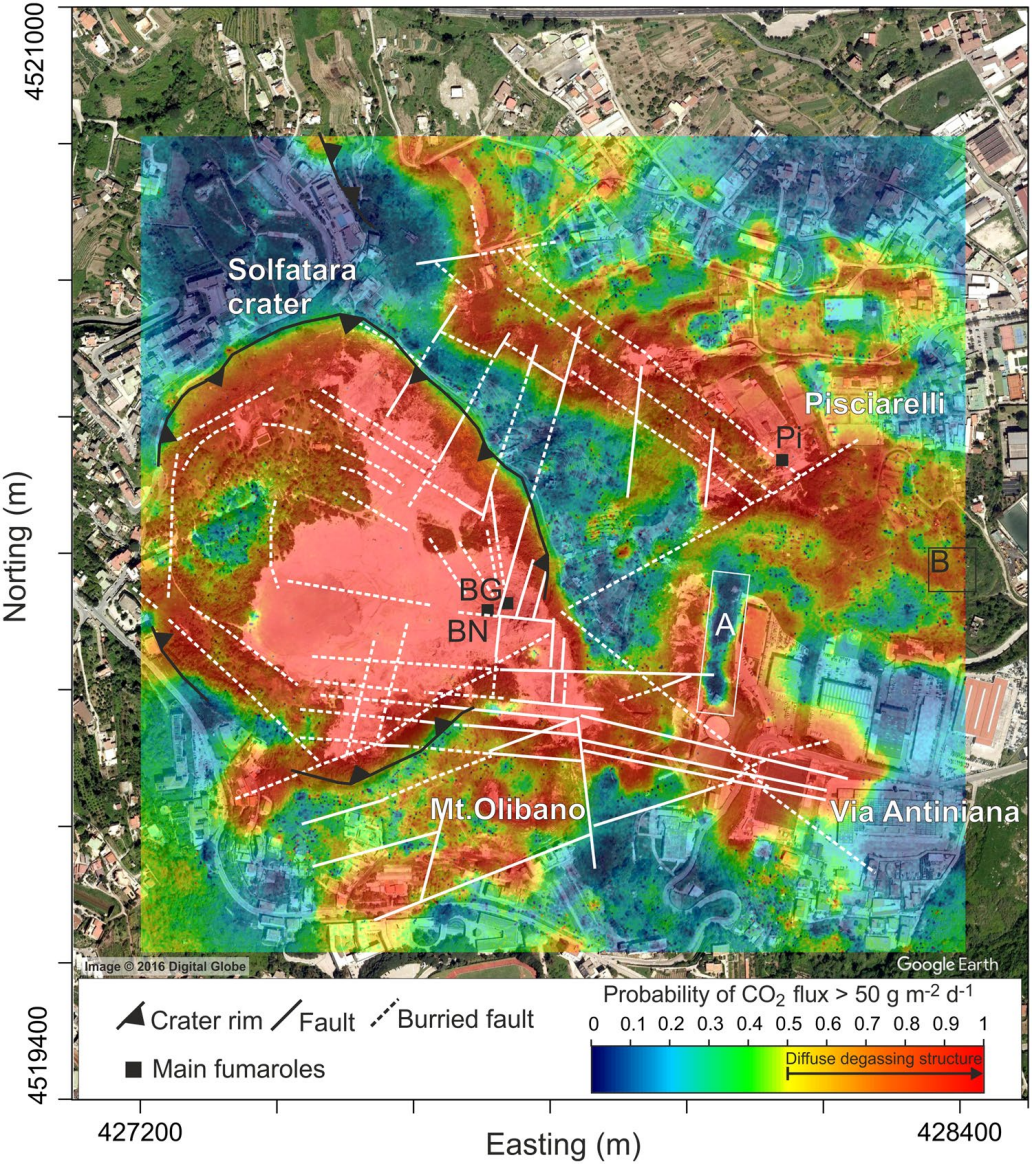
Map of Solfatara diffuse degassing structure (DDS) based on the entire dataset of CO_2_ fluxes from 1998 to 2016. The map was produced by sGs method considering a cell size of 2 × 2 m. The map reports the probability that the simulated CO_2_ flux is greater than 50 g m^−2^ d^−1^, selected as the threshold for a pure biogenic CO_2_ flux, processing the results of 100 simulations (see Methods). Yellow to red colours i.e., probability of CO_2_ flux >50 g m^−2^ d^−1^ higher than 50%, define the Solfatara DDS where degassing of deeply derived CO_2_ occurs. Tectonic structures are from ref. 60. Coordinates are reported as meters projection UTM European Datum 50. The map was created with the software Surfer, Version 11.0.642, (http://www.goldensoftware.com/products/surfer).

To characterise the spatial distribution of CO_2_ fluxes, all 13,158 CO_2_ flux measurements from 1998 to 2016 have been used to produce a map of CO_2_ soil degassing using the sGs approach (see Methods). The result of the sGs simulations is reported as a probability map (Fig. 4) created using the threshold value for the biological background CO_2_ flux as a cut-off. The threshold value selected was 50 g m^−2^ d^−1^, the 95^th^ percentile of the biogenic soil CO_2_ fluxes defined on the basis of the isotopic compositions of the CO_2_ efflux of the March 2007 survey^58^.

The map shows the presence of a well-defined diffuse degassing structure (Solfatara DDS, Fig. 4), that is the area characterised by degassing of deeply derived CO_2_ (see Methods). Solfatara DDS is defined in yellow to red in Fig. 4.

As this map was created considering all the measurements, it reasonably highlights the areas that have been affected more frequently by the degassing of hydrothermal CO_2_ during the last eighteen years.

The Solfatara DDS includes both the area inside the Solfatara crater and neighbouring areas outside the crater, in particular the Pisciarelli area to the E, the Monte Olibano to the S and the area of via Antiniana to the SE (Fig. 4). The shape of the Solfatara DDS is well correlated with the location of volcanic and extensional tectonic structures (faults and fractures, Fig. 4) that allow the gas to transfer towards the surface. The Solfatara DDS is bounded to the NW, and interrupted along a NW-SE band between Solfatara crater and Pisciarelli, by low flux areas corresponding to the outcrop of volcanic products belonging to Astroni deposits^60^ that can act as a permeability barrier for the rising deep CO_2_. In fact, Astroni deposits are locally constituted by massive and fall out and ash surge deposits with thickness from ~10 to ~30 m, affected only by mesoscale normal faults with lengths of tens of centimeters and displacements of a few centimeters^60^.

However, where normal faults deform and cut the Astroni deposits with up to metric dip separation^61^ (e.g., at Via Antiniana area and Pisciarelli), high CO_2_ fluxes occur at the surface (Fig. 4), suggesting that anomalous CO_2_ pressures can be present below this low permeability layer. The roughly NE-SW-elongated CO_2_ flux anomalies in the northern part of Pisciarelli further support the probable continuity of the CO_2_ anomaly below the Astroni deposits. These anomalies match the directions of the drainage network that eroded the Astroni deposits in the eastern flank of the Solfatara cone, resulting in higher fluxes along the valleys. Finally, the geometry of Solfatara DDS is also affected, especially at the Pisciarelli and via Antiniana areas, by intense urbanization (e.g., presence of roads, buildings, excavations and paved squares). In particular, an evident low-flux zone in the via Antiniana area (area A in the map of Fig. 4) coincides with the presence of a paved terrace, while high flux zones in the southern part of Pisciarelli (area B in the map of Fig. 4) correspond to recent excavated zones. This behaviour suggests the high impact of anthropogenic intervention in the natural degassing zones.

**Figure 5:**
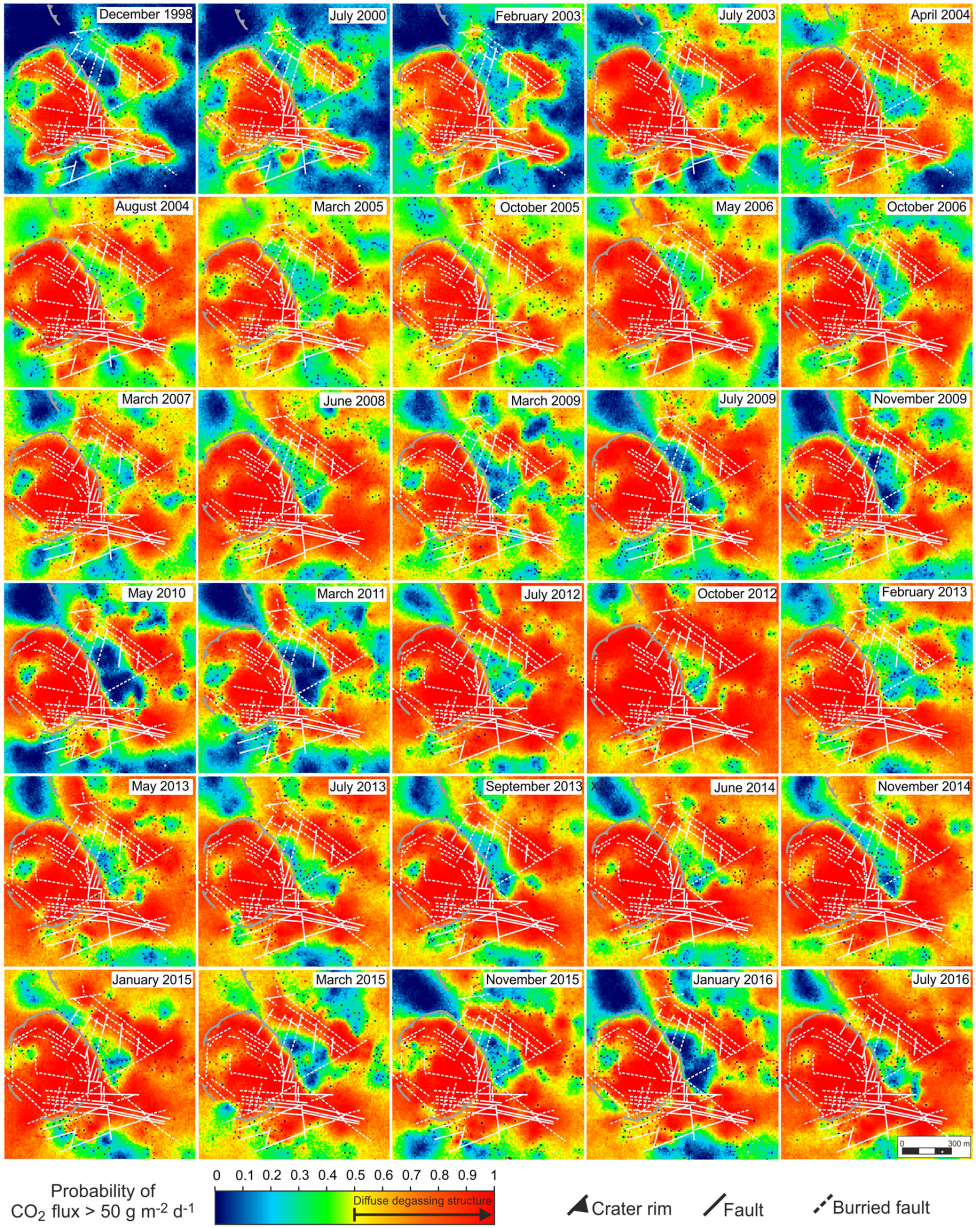
Maps of Solfatara diffuse degassing structure (DDS) in the 1998–2016 period. The maps report the probability that the simulated CO_2_ flux is greater than 50 g m^−2^ d^−1^, selected as the threshold for a pure biogenic CO_2_ flux. Yellow to red colours i.e., probability of CO_2_ Flux >50 g m^−2^ d^−1^ is higher than 50% define the Solfatara DDS where degassing deeply derived CO_2_ occurs. Tectonic structures are from ref. 60. The maps were realized with the software Surfer, Version 11.0.642 (http://www.goldensoftware.com/products/surfer).

To investigate the changes over the time of the spatial distribution of CO_2_ fluxes, and of the total amount of CO_2_ released by diffuse degassing, each dataset was processed by the sGs method. The results are reported in Fig. 5 as probability maps.

The reliability of the produced maps is supported by the good spatial continuity and well-defined spatial structure of all the datasets indicated by experimental variograms of the CO_2_ flux n-score (see Methods, Supplementary Information Fig. S2 and Table S1).

The areal extent of the DDS (1) was computed for each of the maps relating to different surveys (Fig. 5), as the area where the probability that the CO_2_ flux is greater than 50 g m^−2^ d^−1^ is higher than 50% (see Methods). The DDS extent ranges from 0.45 × 10^6^ m^2^ in 1998 to more than 1 × 10^6^ m^2^ in many surveys after 2012. The expansion of DDS mainly interests the areas external to the Solfatara crater, that before 2003 were characterised by low, biogenic, CO_2_ fluxes (Fig. 5). The largest expansion occurred in the Pisciarelli area and particularly in correspondence with the NE-SW fault network of Pisciarelli area and along a band connecting Pisciarelli with the degassing area of via Antiniana in the south. Examples of variations occurred over time in different areas are reported in Supplementary Information Fig. S3. In these areas (areas 1, 2 and 3 in Supplementary Information Fig. S3) the median of the CO_2_ flux passed from typical background values (10–40 g m^−2^ d^−1^) in the pre‐2003 period to values higher up to 1 order of magnitude. The 2003 increase of CO_2_ fluxes and the DDS enlargement were already interpreted as due to the “first arrival” of hydrothermal CO_2_ at the surface, in the peripheral areas of Solfatara^62^.

The total amount of CO_2_ released by diffuse degassing (diffuse total output, DTO) was estimated from the results of the sGs (see Methods) for the different surveys and ranges from 745 (±61) t d^−1^ to 2,815 (±318) t d^−1^ (Table 1). Even if the DTO refers to the sum of all the CO_2_ sources active in the area, it provides a good estimate of the hydrothermal CO_2_ release because CO_2_ fluxes from the hydrothermal source are much higher than those from biogenic sources. For example, assuming a constant biogenic background CO_2_ flux of 26 g m^−2^ d^−1^ over the entire surveyed area, the biogenic CO_2_ output would result in ~38 t d^−1^. Considering this biogenic CO_2_ output for all the surveys, it is only from 1% to 5% of DTO and always within the DTO uncertainty (from 8% to 13%, Table 1). In the following, we will consider the DTO as a representative of the deep hydrothermal source, also considering that the low contribution of biogenic sources is likely an overestimation, as portions of the surveyed area are characterised by the absence of vegetation.

## Discussion

**Figure 6:**
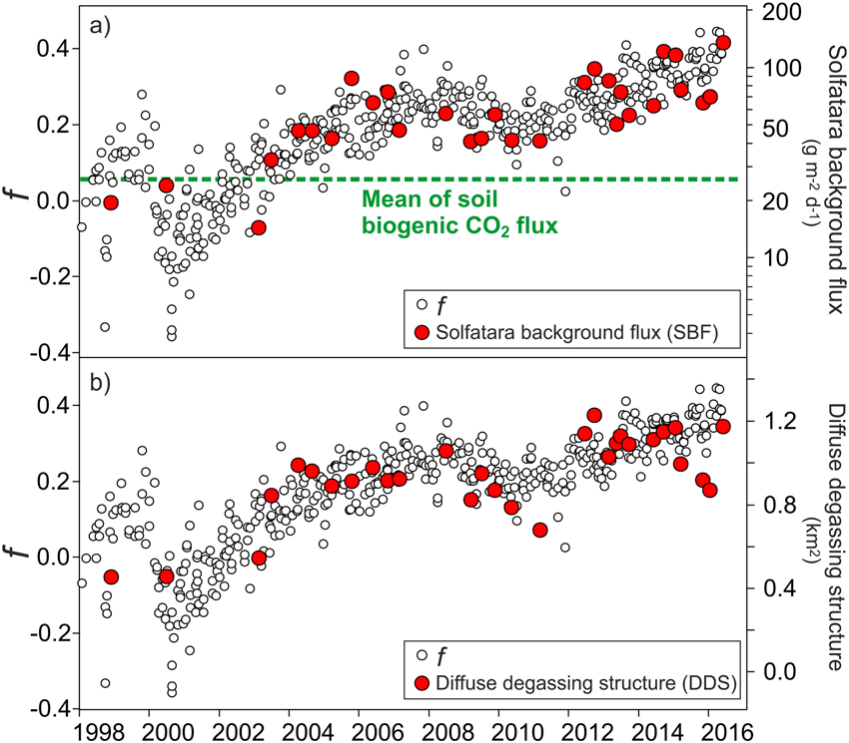
Time variation of the Solfatara background flux (SBF) (**a**) and diffuse degassing structure (DDS) extension (**b**) compared with the fraction of condensation^25^ (*f*) computed for the Solfatara main fumaroles (BG, BN). The variable *f* refers to the fraction of the water removed (sign+) or added (sign−) during the transfer of the gas from the gas equilibration zone to the fumarole (see Methods). The mean of the biogenic CO_2_ flux estimated for Solfatara^58^ is reported as reference in (**a**).

**Figure 7:**
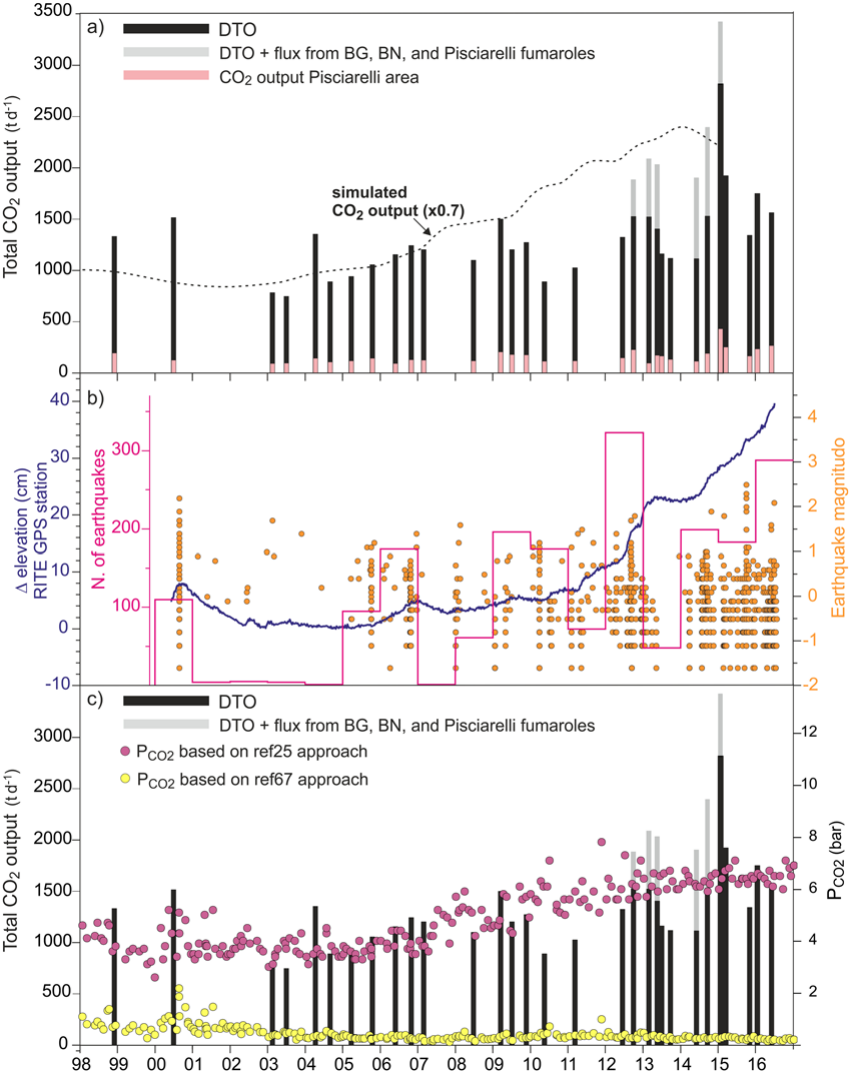
(**a**) Chronogram of the measured CO_2_ release at Solfatara compared with the CO_2_ output resulting from the physical-numerical simulation of ref. 3. Measured CO_2_ flux from the main fumaroles, available since October 2012 and performed in periods not too different from those of CO_2_ flux survey^12, 31, 32, 65^, is reported together with the estimated CO_2_ release by diffuse degassing. The diffuse degassing computed for the Pisciarelli area (area PIS in Fig. 1) is also reported. The CO_2_ output simulated by the physical-numerical simulation is reported with a 0.7 scaling factor. (**b**) Earthquake magnitudes, number of earthquakes and ground elevation at RITE GPS station are reported for comparison^4, 54^ (2016 data update from http://www.ov.ingv.it/ov/it/banche-dati.html). (**c**) Comparison of total CO_2_ output with the P_CO2_ estimations based on two recently published alternative approaches to gas equilibria^25, 67^ (see the text for explanations).

In this section, variations in the CO_2_ degassing during the investigated period are discussed and compared with other geochemical and geophysical signals. We refer in particular to the variations that affected, in one aspect, the “background” CO_2_ emission (LF population) and the extent of DDS (Fig. 6a and b), and in another, the total CO_2_ output (Fig. 7a).

The mean CO_2_ flux of the LF population, which will be referred here as SBF (Solfatara background flux), increases from 20–30 g m^−2^ d^−1^ in 1998 to 136 g m^−2^ d^−1^ in the last campaign of June 2016, following a scattered, but almost continually increasing trend (Fig. 6a).

It is worth noting that typical mean CO_2_ fluxes generated by a biogenic source in the soil (20–30 g m^−2^ d^−1^, see above) were only measured in the first three campaigns, whilst from 2003 onwards, SBF were 2–5 times higher. This increase implies that after 2003, the SBF began to represent a mixture of biogenic and deeply-derived CO_2_, indicating that areas previously unaffected by an anomalous CO_2_ degassing started to release deeply-derived gas. In fact, the SBF increase is correlated with an important change of the spatial pattern of CO_2_ fluxes as shown by maps of the Solfatara DDS (Fig. 5). Chronograms in Fig. 6 show an initial growth of Solfatara DDS and an increase of SBF from February 2003 to March 2004, when the DDS doubled its extent from ~0.5 km^2^ to ~1 km^2^ (Table 1 and Fig. 6b) and SBF increased from 20–30 g m^−2^ d^−1^ to 40–50 g m^−2^ d^−1^. After this expansion, the DDS extent and the SBF remained quite stable until July-October 2012 when, after a relative reduction, DDS reached an extent of ~1.2 km^2^ (Table 1) and SBF increased to 70–100 g m^−2^ d^−1^. We observe that the DDS extent reached 80–90% of the 1.4 km^2^ of the investigated area, implying that this parameter is now close to saturation and further important variation in the future are not possible while SBF could continue to increase.

The SBF increase and the extension of Solfatara DDS may reflect variations in the vapor plume (Fig. 2) feeding the Solfatara and Pisciarelli emissions. According to ref. 25, steam condensation and temperature increase affects the hydrothermal system of Solfatara. This process could lead to a generalised increase of fluid pressure and to the formation of batches of condensates within the vapor plume^25^. Repeated episodes of mud emission^3, 25^ at Pisciarelli, occurred in April 2006, July 2012, October 2013, July 2014, February 2015, May 2016 and February 2017, confirm the increased amount of condensates produced by the system. It is worth noting that the enlargement of the Solfatara DDS and the increase of SBF proceed concurrently with the increase in the fraction *f* of condensation estimated from the composition of the main fumaroles of Solfatara (Fig. 6a and b, see Methods). Therefore, it is highly probable that the SBF increase, as well as the enlargement of Solfatara DDS, is linked to the deep dynamics of the vapor plume and in particular to the ongoing condensation and heating processes.

Despite the good agreement between variations of DDS, SBF and *f* values, deviations from the long-term trend depicted in Fig. 6 can be caused by a short-term process and by the uncertainty of *f* estimation. For example, the DDS extent could be partially controlled by the rain that affect CO_2_ fluxes increasing soil water content, changing soil permeability, dissolving soil CO_2_, etc. These processes can affect the DDS extent because are more effective in low-flux areas, that are those mainly contributing DDS extent variations.

Other relevant changes regarded the DTO (Fig. 7), that is well representative of deep source degassing.

The DTO measured during the first two campaigns (1998 and 2000) is relatively high (1,300–1,500 t d^−1^) while the two following campaigns, performed in 2003, are characterised by the lowest values (750–800 t d^−1^). Since then, a trend of increasing DTO begins, accompanying the ongoing unrest of CFc. The highest CO_2_ fluxes were measured in the last six campaigns (after September 2014) when DTO values, excluding November 2015, are above 1,500 t d^−1^ and reach peak values of 2,800 t d^−1^ and 1,900 t d^−1^ in January and March 2015, respectively.

The increase of DTO is mainly due to the increase of the degassing rate in areas of high CO_2_ flux that reached a peak in 2014–2015. For example, considering the measured fluxes inside the Solfatara crater and in the fumarolic area southern of BG-BN flumaroles (areas 4 and 5 in Supplementary Information Fig. S3) the median values of CO_2_ flux increased from ~500 g m^−2^ d^−1^ and ~400 g m^−2^ d^−1^ during 2003–2013 to ~800 g m^−2^ d^−1^ and ~1,100 g m^−2^ d^−1^ after 2013, respectively (Supplementary Information Fig. S3). The most important increase occurred in Pisciarelli area where the CO_2_ flux passed progressively from ~100 g m^−2^ d^−1^ to ~300 with a peak value of ~1,000 g m^−2^ d^−1^ in 2015.

The relative increase of CO_2_ fluxes is particularly relevant at Pisciarelli, where the CO_2_ diffuse emission rose from 90 t d^−1^ to 260 t d^−1^ from 2003 to 2016, with a large peak of 428 t d^−1^ in 2015 (area PIS in Fig. 1). Furthermore, at Pisciarelli, the increase of diffuse emission corresponds to an evident macroscopic intensification of the hydrothermal activity^25, 53^.

The DTO represents only a fraction of the total CO_2_ output, as it does not include the contribution from fumarolic vents. The vent contribution to the Solfatara CO_2_ budget most probably increased in the last years concurrently with the abovementioned increase in the fumarolic activity. Measurements of the CO_2_ flux performed in the 1980’s (refs 63 and 64) suggest, in fact, that the CO_2_ emission from fumaroles was relatively low in the past, also during the large crisis of 1982–1984. On the contrary, more recent measurements (2012–2015 period) performed with different techniques point to significant CO_2_ emissions from the fumarolic vents that, from BN-BG and Pisciarelli fumaroles, were estimated to be 343 to 858 t d^−1^ (refs 12, 31, 32 and 65). It is also worth noting that the CO_2_ flux from the vents of Pisciarelli area, that was evaluated at only ~18 t d^−1^ in March 2009, reaches values of 170 t d^−1^ to 490 t d^−1^ in 2012–2015 (ref. 31). Finally, a high total (diffuse + vents) CO_2_ flux of 2,300 to 4,600 t/d from the Solfatara crater was estimated in March 2016 based on a new portable DIAL laser system, while the same method applied at Pisciarelli gave 266 ± 211 t d^−1^ (ref. 33).

In Fig. 7b the CO_2_ release is compared with the seismicity^4^ and ground deformation^54^. Since 2005, the occurrence of earthquakes (located at depth <3 km, ref. 66) increased together with the beginning of an uplift phase. Since 2005, a general correlation emerges between the increase of CO_2_ release and the increase in seismicity and ground uplift, although the gas flux time series is more discontinuous than geophysical ground uplift and seismicity records (Fig. 7b). In particular, in the period from 2012 to 2016, two main peaks in the CO_2_ output coincide with episodes of accelerating ground uplift and of intensification in the seismicity. It is also worth noting, that these two periods of stronger degassing are separated by a period of relatively low CO_2_ output, which corresponds to a period of low seismicity and a pause in the deformation.

These correlations support the link between the degassing and the deformation-seismicity, both controlled by repeated episodes of magmatic fluids input into the hydrothermal system, episodes that increased in frequency after 2005 (refs 3, 4 and 25). These episodes of magmatic fluid injections, modelled with a geothermal simulator, involved increasing amounts of fluids since the mid 2000s^3, 4^. Here we show, for the first time, the modelled CO_2_ output returned by the last published simulation of the process^3^. The modelled CO_2_ output from the entire simulation domain (Fig. 2) shows roughly the same increasing trend of the measurements (Fig. 7a), even if the observed CO_2_ fluxes are systematically lower than the modelled CO_2_ output (~70%). Considering the approximations of the model, this discrepancy is very low. It is possibly caused by either a general overestimation in the modelling, or the fact that our measurements do not include the entire amount of the released CO_2_. For example, the measurements neither include the contribution of the many low flux fumaroles (which are not measured and not measurable with the AC, see Method), nor the CO_2_ emissions from zones outside the investigated area. In any case, the match between modelled emission and measurements provides convincing evidence that the visible escalation in degassing activity is caused by repeated injections of magmatic gases into the Campi Flegrei hydrothermal system as simulated in the modelling^3^.

Recently, two alternative approaches to gas equilibria leaded to opposite results regarding the temporal evolution of T-P conditions of the hydrothermal system feeding the Solfatara emission. Ref. 25 points to a generalized increase of pressures and temperatures while the other interpretation indicates the progressive depressurization of the system^67^, a process that would be still ongoing. This depressurization is clearly in contrast with the evolution of total CO_2_ output that almost tripled from early 2000’s in agreement with the increase of the pressure in the hydrothermal system as returned by the model of ref. 25 (Fig. 7c).

The CO_2_ total output in recent years from the Solfatara hydrothermal system can be reasonably assumed to be at least 2000–3000 t d^−1^. For comparison, the estimations for the last period are much higher than the CO_2_ emitted by geothermal power plants around the world. They are, for example, 4–6 times higher than those emitted by Icelandic geothermal power plants (435 t d^−1^, ref. 68) and ~3 times more than those associated with geothermal plants in New Zealand (~800 t d^−1^; www.nz.geothermal.org.nz/emissions/).

**Figure 8:**
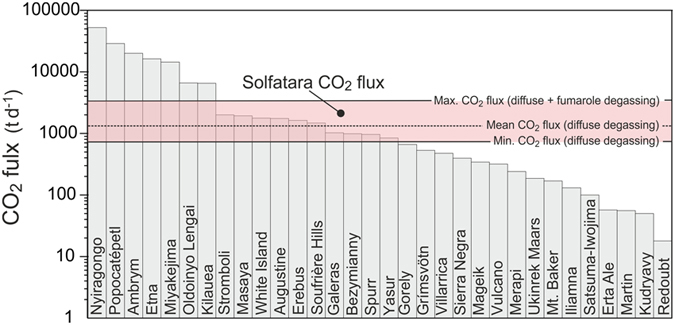
CO_2_ fluxes from Solfatara compared to mean volcanic plume CO_2_ fluxes from persistently degassing volcanoes (data from ref. 69). For Solfatara, the minimum and average CO_2_ flux from diffuse degassing measured for the 1998–2016 period and the maximum CO_2_ flux resulting from the sum of diffuse degassing and fumaroles fluxes in the period 2012–2016 are reported.

In Fig. 8, the CO_2_ flux released by Solfatara is compared with the mean volcanic plume CO_2_ fluxes from persistently degassing volcanoes reviewed by ref. 69. Considering only the contributions from soil diffuse degassing, it emerges that Solfatara DDS on average (i.e., mean DTO = 1,309 t d^−1^) sustains a daily CO_2_ flux to the atmosphere similar to a “medium-large” volcanic plume. If, instead, we consider the highest total CO_2_ release (vents + diffuse) of 3,420 t d^−1^, measured in January 2015, this would constitute the eighth highest value ever measured at volcanic plumes.

This finding queries the reliability of the actual estimates of the natural flux of CO_2_ from volcanic activity, considering that many calderas around the world which are affected by hydrothermal sites, similar to Campi Flegrei, are not normally included in most of the global estimates. We think that the flux of CO_2_ from hydrothermal sites is potentially globally relevant considering also that can reach very high values up to the 10–60 kt d^−1^ estimated for Yellowstone^17, 70^.

The long-term volcanic CO_2_ degassing at Solfatara, and degassing from hydrothermal systems in general, can contribute to refining estimates of the volcanic CO_2_ contribution to the atmosphere, and can aid the ability to assess the possible role of CO_2_ degassing from hydrothermal systems.

## Methods

### CO_2_ flux measurement

CO_2_ flux measurements were performed using the accumulation chamber method, which is based on the measurement of the CO_2_ concentrations over time, inside an inverted chamber placed on the ground^27–29^. The used instruments were developed, assembled and tested at the laboratories of Università di Perugia and INGV of Naples. Each instrument consists of: (1) a metal cylindrical vessel (the accumulation chamber, AC), (2) an Infra-Red (IR) spectrophotometer, (3) an analog-digital (AD) converter, and (4) a palmtop computer (Supplementary Information Fig. S4). Since 2003, the measuring apparatuses have been equipped with LI-COR IR sensors, Li-800 or LICOR Li-820, operating in the range 0–20,000 ppm of CO_2_. The instrument used for the surveys of 1998 and 2000 was equipped with a Dräeger Polytron IR sensor with adjustable measurement range from 2,000 ppm up to 100% vol. The gas is circulated from the AC, of ~2.8 L volume, to the IR and vice versa by a pump (~0.0167 L s^−1^). The AC is internally equipped with ring-shaped perforated manifold that re-injects the gas favouring the mixing in the chamber. The CO_2_ concentration in the circulating gas is acquired every 0.025 s and transmitted to a palmtop computer where is plotted versus the measuring time. A specific applicative (Gasdroide, https://bitbucket.org/moovida/gasdroide), capable of acquiring the digital signals from the AD and to elaborate concentration vs time diagram, was developed in 2012 for the Android operating system and released with a General Public License.

The CO_2_ flux is computed in real time from the rate of CO_2_ concentration increase in the chamber (dC_CO2_/dt) according to the relation CO_2_ flux = *cf* × dC_CO2_/dt (ref. 28). The proportionality factor (*cf*) between dC_CO2_/dt and the CO_2_ flux was determined before each survey by laboratory tests, during which imposed CO_2_ fluxes typically in the range 10 to 10,000 g m^−2^ d^−1^, were measured over a “synthetic soil” made of dry sand (10 cm-thick) placed inside a plastic box. In each calibration test, the *cf* value was computed from the linear best-fit line of CO_2_ flux vs. dC_CO2_/dt.

The measured soil CO_2_ flux and the measurement locations of each survey are reported in Supplementary Information Dataset S1, together with the soil temperature at ~10 cm depth measured at the same time and at the same site of CO_2_ flux measurement. The soil temperature data is provide for completeness but is not discussed in this work.

### Statistical data elaboration

The CO_2_ flux data was analysed by statistical methods to define the statistical parameters of the flux, which offer insight into the origin of degassed CO_2_.

In volcanic-hydrothermal areas, the CO_2_ soil diffuse degassing is frequently fed by multiple gas sources, such as biological and volcanic (ref. 29 and references therein). The multiple origin of the gas can result in a bimodal statistical distribution of CO_2_ flux values, which plots as a curve with an inflection point on a logarithmic probability plot (see Fig. 3). In fact, while a single log-normal population plots as a “straight” line on a logarithmic probability plot, a polimodal distribution resulting from the overlapping of n log-normal populations plots as a curve with n-1 inflection points^71^. The partition of such complex statistical distributions into individual log-normal populations and the estimation of the proportion (*f*_i_), the mean (*M*_i_), and the standard deviation of each population were performed following the graphical-statistical procedure proposed by ref. 71. which is largely applied to soil CO_2_ fluxes^28, 29, 57^. Since the so computed *M*_*i*_ value refers to the logarithm of the CO_2_ flux values, the mean value of CO_2_ flux was estimated using a Montecarlo simulation procedure (Table 1).

The estimated mean CO_2_ flux values have been used in literature to compute the CO_2_ output pertinent to each population associating a fraction of the degassing area (i.e., *S*_*i*_ = *f*_*i*_ *S* where *S* is the total extent of the surveyed area, GSA approach)^28^. However, the reliability of the CO_2_ output obtained from the GSA can be affected by arbitrary choices in the partitioning procedure^29^. In particular, the interpretations of the tails of the distributions are critical as, especially at the high flux values, they are generally defined based on a low number of measured values. Furthermore, the GSA approach does not consider the spatial distribution of the measurements implicitly assuming a homogeneous sampling density.

### Geostatistical data elaboration

In order to obtain a reliable estimation of the total CO_2_ output and to produce maps of the CO_2_ flux, the CO_2_ fluxes were elaborated with a geostatistical approach proposed by ref. 29 based on sequential Gaussian simulations (sGs). According to refs 29 and 72, sGs yields a realistic representation of the spatial distribution of CO_2_ fluxes reproducing both the statistics and the spatial features of the experimental data.

The sGs method consists of the production of numerous equiprobable realizations of the spatial distribution of an attribute (i.e., maps of CO_2_ flux in this study), here performed using the *sgsim* algorithm of the GSLIB software library^73^. Since the sGs assumes multi-Gaussian distribution of the attribute to be simulated, the CO_2_ flux values were transformed into a normal distribution (n-scores of data) using the *nscore* algorithm of the GSLIB software library. The n-scores are then used in the simulation procedure and transformed back into flux values at the end of the simulation process, using the inverse of the normal score transform. The CO_2_ flux values are simulated at locations defined by a regular grid, here consisting in a grid of 14,520 squared cells (121 cells in the EW and 120 cells in the NS direction) with a cell size of 10 × 10 m (except for the map reported in Fig. 2 for which a 2 × 2 m simulation cell was used). The n-scores are simulated by random sampling of a Gaussian cumulative distribution function (cdf), defined at each location on the basis of a mean value and variance computed at each grid node by means of simple kriging method. Simple kriging estimate and variance are computed considering the measured data and those previously simulated during the procedure, according to the variogram model of n-scores and to the statistical distribution of the data. The variogram model is defined fitting the experimental variogram of n-scores and provides a description of how the data are spatially correlated. The variogram models are given in terms of nugget, range and sill parameters, where the nugget represents the small-scale variation (including measurement errors), the range represents the distance within which data are correlated and the sill is the plateau the variogram reaches for a distance equal to the range. The simulation was run in order to produce 100 realizations for each dataset.

The produced realizations were post-processed to produce the E-type estimate map and the probability map. The E-type estimate map, i.e. the map of the “expected” value at any location, is obtained through a pointwise linear average of all the realizations. The probability map consists in a map of the probability that, among all the realizations, the simulated CO_2_ flux at any location (i.e., at grid nodes) is above a cut-off value. The probability map drawn for each survey is reported in Fig. 5 while the maps of E-type estimate are reported in the Supplementary Information Fig. S5.

According to ref. 29, selecting the threshold value of biogenic CO_2_ flux as a cut-off, the probability maps were used to define the extent of the diffuse degassing structures (DDS)^28^, that is the area interested by the release of deeply derived CO_2_. According to ref. 29 in this work, the DDS is considered as the area where the probability, among the 100 realizations, that the simulated CO_2_ flux is higher than the biogenic CO_2_ flux threshold over 50%.

The simulated flux values by sGs were used also to compute the total CO_2_ release by diffuse degassing. The total CO_2_ release is computed for each realization by summing the products of the simulated CO_2_ flux value at each grid cell by the cell surface. The mean of the values of total CO_2_ release computed for the 100 realizations are assumed as the characteristic diffuse total CO_2_ output (DTO, see above) for each period. The standard deviation of the 100 estimates is assumed as the DTO uncertainty.

### Fraction of condensed steam

A previous work by ref. 25 illustrated several evidences of heating of the hydrothermal system feeding the Solfatara emissions based on the compositional variations of the main fumaroles of the area (BG and BN). The pressurization of the vapor plume (Fig. 2), that in turn causes the condensation of the steam^25^, would cause the heating of the system. The occurrence of condensation-heating was successively returned by a physical modelling approach^3^. The ref. 25 proposed a method to compute the fraction of the condensed steam starting from the fumarolic compositions. Because the computation in the original work is not detailed, here we illustrate the mass balance on which the computation is based on. The mass balance of a batch of gas which passes from reservoir to fumaroles, can be expressed in terms of number of moles of CO_2_ and H_2_O in the reservoir (*n*_*CO2*,*eq*_, *n*_*H2O*,*eq*_), in the fumarole (*n*_*CO2*,*fu*_, *n*_*H2O*,*fu*_) and the number of moles of water removed (*n*_*H2O*,*rem*_) and/or added (*n*_*H2O*,*add*_) during the fluid transfer. Assuming that secondary processes do not affect CO_2_, the mass balance equations are:

1 $${n}_{C{O}_{2},eq}={n}_{C{O}_{2},fu}$$

2 $${n}_{{H}_{2}O}={n}_{{H}_{2}O,fu}+{n}_{{H}_{2}O,rem}-{n}_{{H}_{2}O,add}$$

We can express the number of moles of water removed and/or added as the product of a proportionality factor (*f*_*rem*_, *f*_*add*_) by the original number of moles (*n*_*H2O*,*eq*_):

3 $${f}_{rem}\times {n}_{{H}_{2}O,eq}={n}_{{H}_{2}O,rem}$$

4 $${f}_{add}\times {n}_{{H}_{2}O,eq}={n}_{H{}_{2}O,add}$$

Inserting equations (3) and (4) in equation (2), we obtain:

5 $$\begin{array}{rcl}{n}_{{H}_{2}O,eq} & = & {n}_{{H}_{2}0,fu}+f{}_{rem\,}\times {n}_{{H}_{2}O,eq}-f{}_{add\,}\times {n}_{{H}_{2}O,eq}\\  & = & n{}_{{H}_{2}O,fu\,}+({f}_{rem}-{f}_{add})\times {n}_{{H}_{2}O,eq}\\  & = & n{}_{{H}_{2}O,fu\,}+f\times {n}_{{H}_{2}O,eq}\end{array}$$

where *f* = *f*_*rem*_ − *f*_*add*_ is positive in the case of water removal and negative in the case of water addition. Dividing equation (5) by equation 1 gives:

6 $$\frac{{n}_{{H}_{2}O,eq}}{{n}_{C{O}_{2},eq}}=\frac{{n}_{{H}_{2}O,fu}}{{n}_{C{O}_{2},fu}}+f\times \frac{{n}_{{H}_{2}O,eq}}{{n}_{C{O}_{2},fu}}$$

Considering that *n*_*CO2*,*fu*_ = *n*_*CO2*,*eq*_, equation (6) can be written as:

7 $$\frac{{n}_{{H}_{2}O,eq}}{{n}_{C{O}_{2},eq}}=\frac{{n}_{{H}_{2}O,fu}}{{n}_{C{O}_{2},fu}}+f\times \frac{{n}_{{H}_{2}O,eq}}{{n}_{C{O}_{2},eq}}$$

From equation (7), the variable f is given by:

8 $$f=\frac{\frac{{n}_{{H}_{2}O,eq}}{{n}_{C{O}_{2},eq}}-\frac{{n}_{{H}_{2}O,fu}}{{n}_{C{O}_{2},fu}}}{\frac{{n}_{{H}_{2}O,eq}}{{n}_{C{O}_{2},eq}}}$$

Finally, equation (8) is solved considering $\frac{{n}_{{H}_{2}O,eq}}{{n}_{C{O}_{2},eq}}=\frac{{X}_{{H}_{2}O,eq}}{{X}_{C{O}_{2},eq}}=\frac{{P}_{{H}_{2}O,eq}}{{P}_{C{O}_{2},eq}}$ and $\frac{{n}_{{H}_{2}O,fu}}{{n}_{C{O}_{2},fu}}=\frac{{X}_{{H}_{2}O,fu}}{{X}_{C{O}_{2},fu}}$:

9 $$f=\frac{\frac{{P}_{{H}_{2}O,eq}}{{P}_{C{O}_{2},eq}}-\frac{{X}_{{H}_{2}O,fu}}{{X}_{C{O}_{2},fu}}}{\frac{{P}_{{H}_{2}O,eq}}{{P}_{C{O}_{2},eq}}}$$

The variable X_CO2,fu_ and *X*_*CO2*,*fu*_ are the analytical values, while *P*_*H2O*,*eq*_ and *P*_*CO2*,*eq*_ refer to the equilibrium values computed considering gas equilibria in the H_2_O-H_2_-CO_2_-CO gas system. In detail, we used the following expressions^38, 74^:

10 $$T(K)=\frac{3133.5}{0.933-\,\mathrm{log}\,\frac{{X}_{CO}}{{X}_{C{O}_{2}}}}$$

11 $$\mathrm{log}\,{P}_{{H}_{2}O}=5.51-\frac{2048}{T(K)}$$

12 $$\mathrm{log}\,{P}_{C{O}_{2}}=-2.485+\frac{2248}{T(K)}-\,\mathrm{log}\,\frac{{X}_{{H}_{2}}}{{X}_{CO}}+\,\mathrm{log}\,{P}_{{H}_{2}O}$$

where the partial pressures of water and CO_2_ are assumed equal to their fugacities.

Equation (9) was used to compute the condensed fraction *f* for each BG and BN fumarolic samples reported in Fig. 6. The fumarolic compositions are available in the literature^3^.

## Electronic supplementary material

Supplementary Information

Dataset 1
