# Supplementary material for: Monitoring diffuse volcanic degassing during volcanic unrests: the case of Campi Flegrei (Italy)
Source: Sci Rep. 2017 Jul 28;7:6757. doi: 10.1038/s41598-017-06941-2 (PMC5533770; doi:10.1038/s41598-017-06941-2)
Supplement: Supplementary file 1 — Supplementary Information [file 41598_2017_6941_MOESM1_ESM.pdf]

Supplementary Information

Monitoring diffuse volcanic degassing during volcanic unrests: the case of Campi Flegrei (Italy).

Cardellini C.[1], Chiodini G.[2], Frondini F.[1], Avino R. [3], Bagnato E. [1], Caliro S. [3], Lelli M.[4], Rosiello A.[1]

1) Dipartimento di Fisica e Geologia, Università degli studi di Perugia, via Pacoli snc, 06123 Perugia, Italy.
2) Istituto Nazionale di Geofisica e Vulcanologia, sezione di Bologna, via D. Creti 12, 40128 Bologna, Italy.
3) Istituto Nazionale di Geofisica e Vulcanologia, sezione di Napoli Osservatorio Vesuviano, via Diocleziano 328, 80124 Napoli, Italy.
4) Consiglio Nazionale delle Ricerche, Istituto di Geoscienze e Georisorse, Via G. Moruzzi 1, 56124 Pisa, Italy.

Supplementary Figure S1

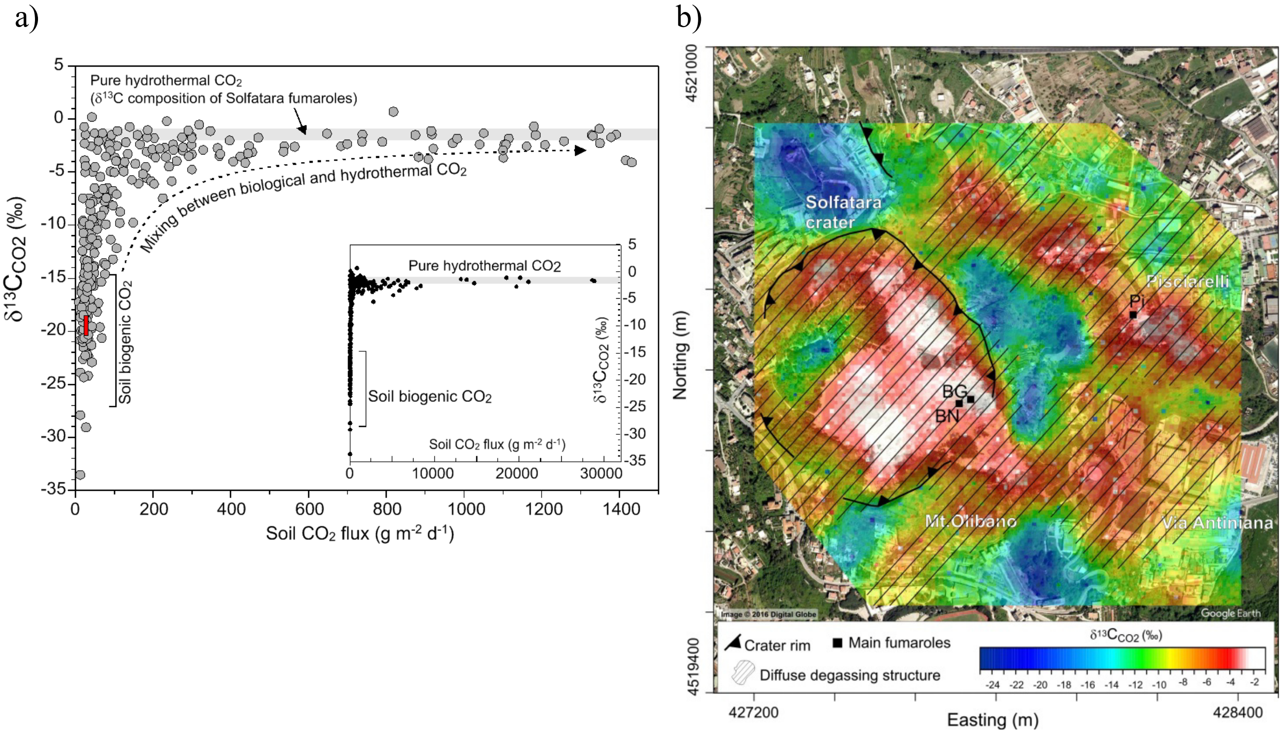

Figure S1. Soil CO_2 flux and CO_2 efflux isotopic composition of March 2007 survey. a) Diagram of soil CO_2 flux and CO_2 efflux isotopic composition. The grey band reports the range of CO_2 isotopic composition of Solfatara fumaroles in the period 2005-2008 (ref.1) and the red rectangle the estimated mean flux and isotopic compositions of the biogenic CO_2 (ref.2). The range of soil biogenic CO_2 (ref.3) is also reported. At high CO_2 fluxes the isotopic composition of the CO_2 efflux matches the isotopic composition of fumaroles CO_2 (inset), while CO_2 fluxes < 200 g m^{-2} d^{-1} the isotopic composition of the efflux results from the mixing between biogenic CO_2 with variable amounts of hydrothermal CO_2. b) Map of CO_2 efflux isotopic composition of March 2007. In the map is also reported the area of the Solfatara diffuse degassing structure (DDS, dashed area) corresponding to area where the probability of CO_2 flux > 50 g m^{-2} d^{-1} is higher than 50% (see Methods). The map was realized with the software Surfer, Version 11.0.642 (http://www.goldensoftware.com/products/surfer)

References

1. Chiodini, G. *et al.* Long-term variations of the Campi Flegrei, Italy, volcanic system as revealed by the monitoring of hydrothermal activity. *J. Geophys. Res* **115** (2010).
2. Chiodini, G. *et al.* Carbon isotopic composition of soil CO_2 efflux, a powerful method to discriminate different sources feeding soil CO_2 degassing in volcanic-hydrothermal areas. *Earth Planet. Sci. Lett.* **274**, 372-379 (2008).
3. Cheng, W. Measurement of rhizosphere respiration and organic matter decomposition using natural ^{13}C. *Plant Soil* **183**, 263-268 (1996)

Supplementary Figure S2

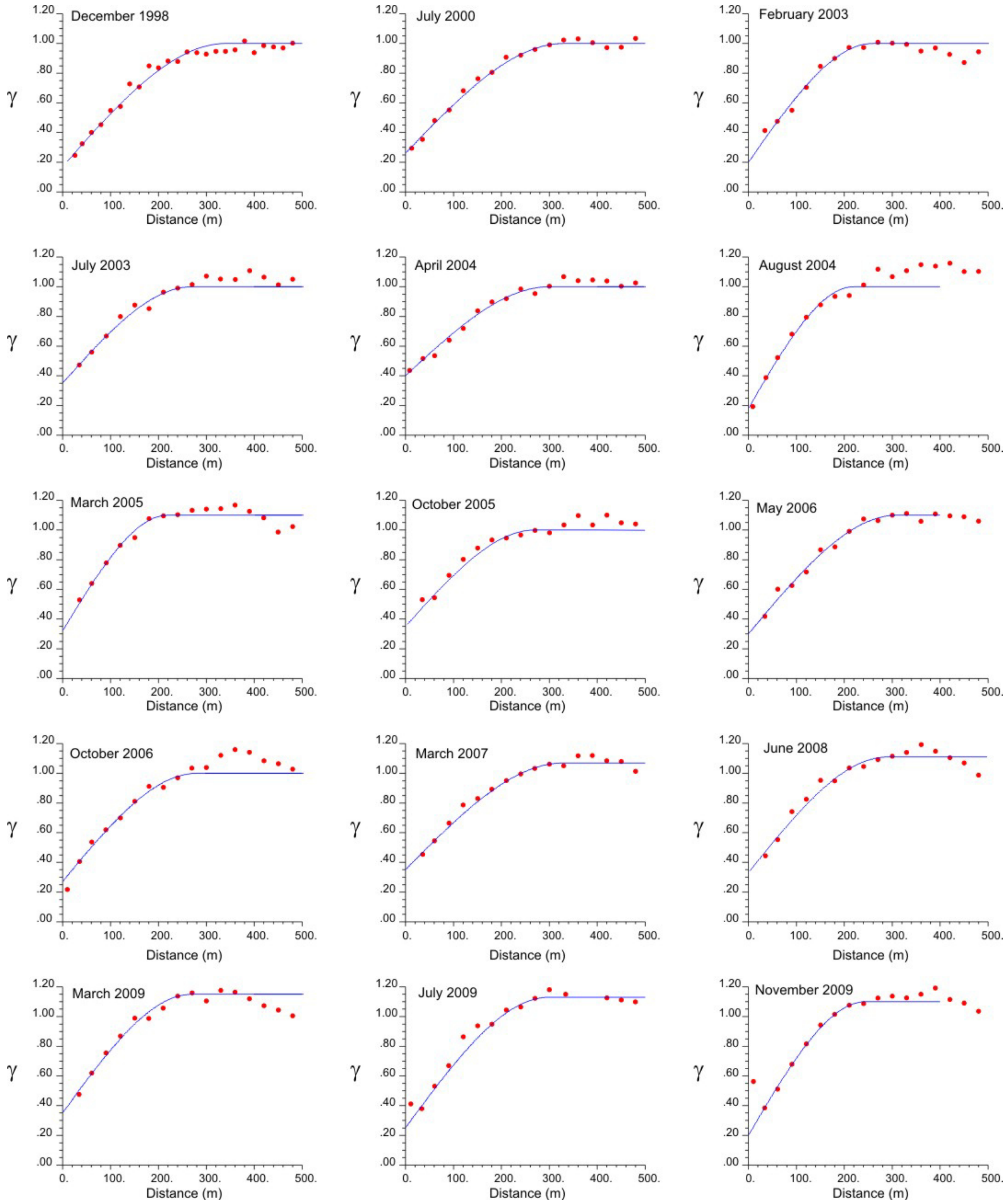

(continue in the next page)

Supplementary Figure S2 (continue)

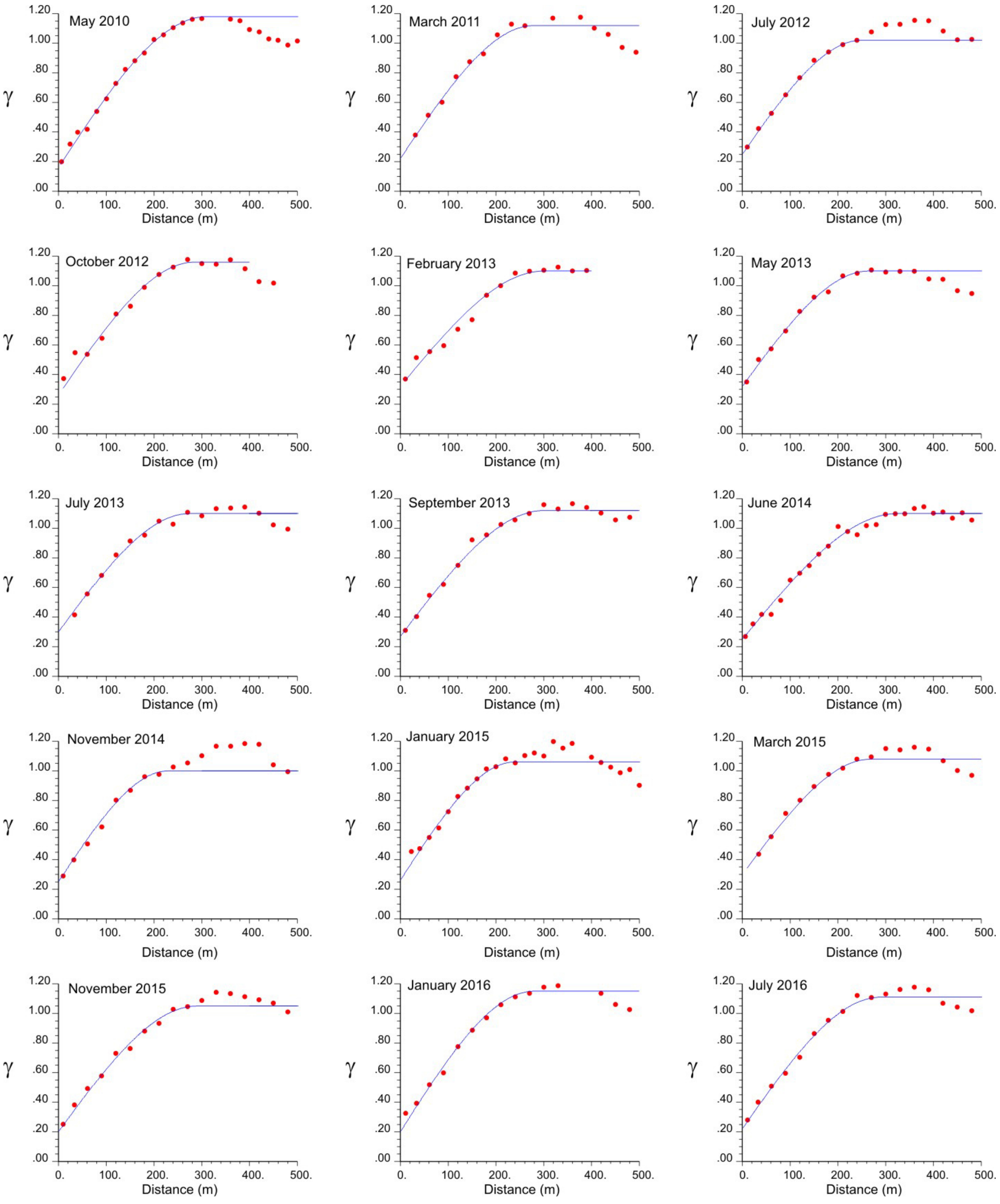

Figure S2. Experimental variograms of CO_2 flux n-scores of each dataset and variogram models. In the figure are reported the experimental variograms with red dots and the compute variogram models (blue lines, see Table S1) that were used to produce the CO_2 flux maps with the sGs method.

Supplementary Figure S3

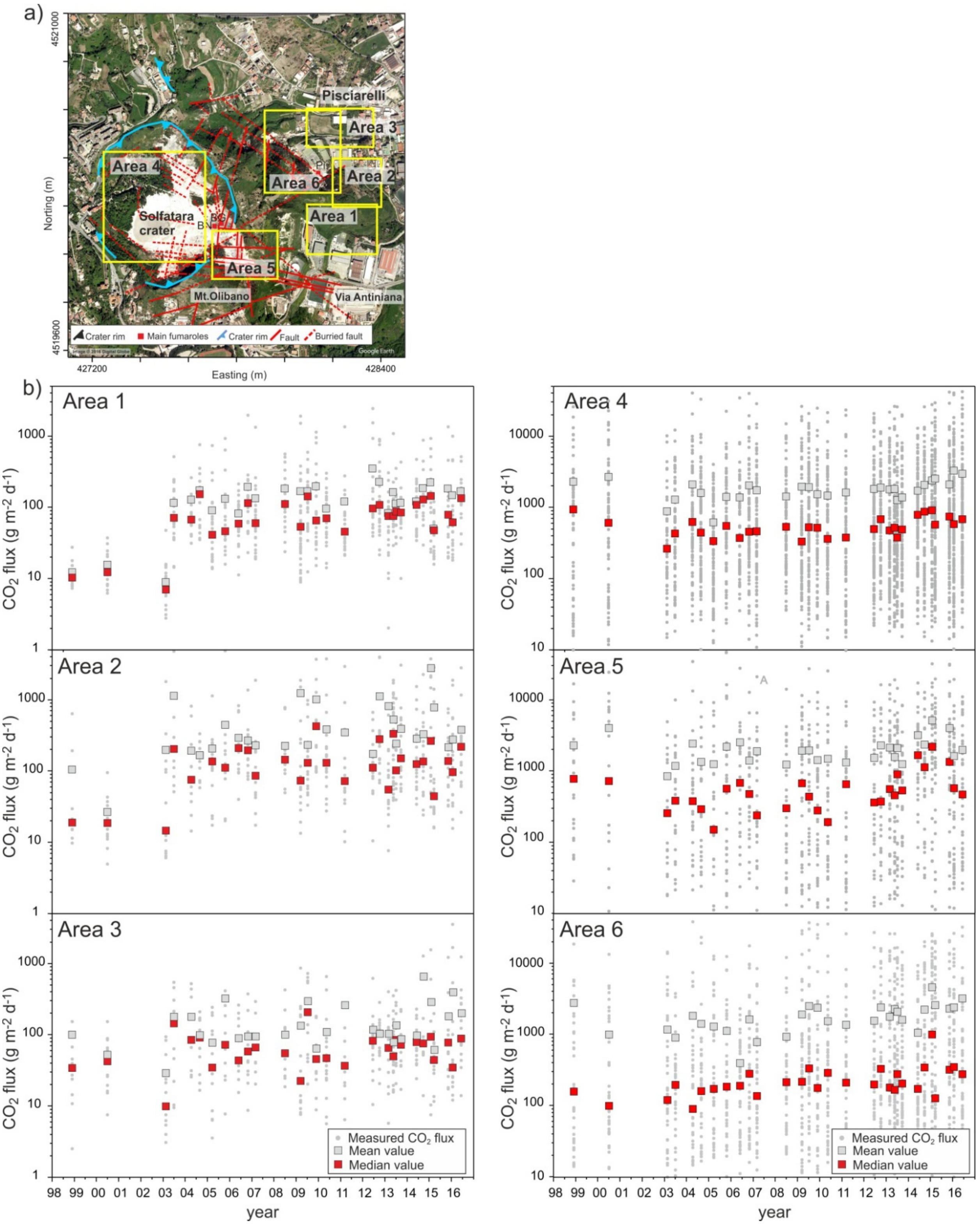

Figure S3. **Time variation of measured CO_2 fluxes in several areas of the study area**. a) Sketch map with the location of the areas considered. The map was realized with the software Surfer, Version 11.0.642 (http://www.goldensoftware.com/products/surfer). b) Diagram of time variation of measured CO_2 flux in the different areas. The mean value and the median value are reported for each dataset.

Supplementary Figure S4

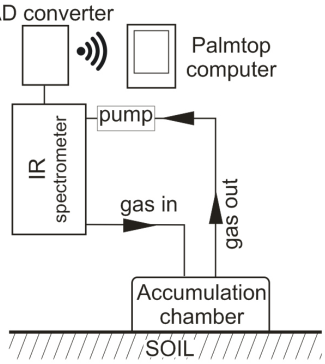

Figure S4. Sketch of the accumulation chamber apparatus used for the CO_2 flux measurements

Supplementary Figure S5

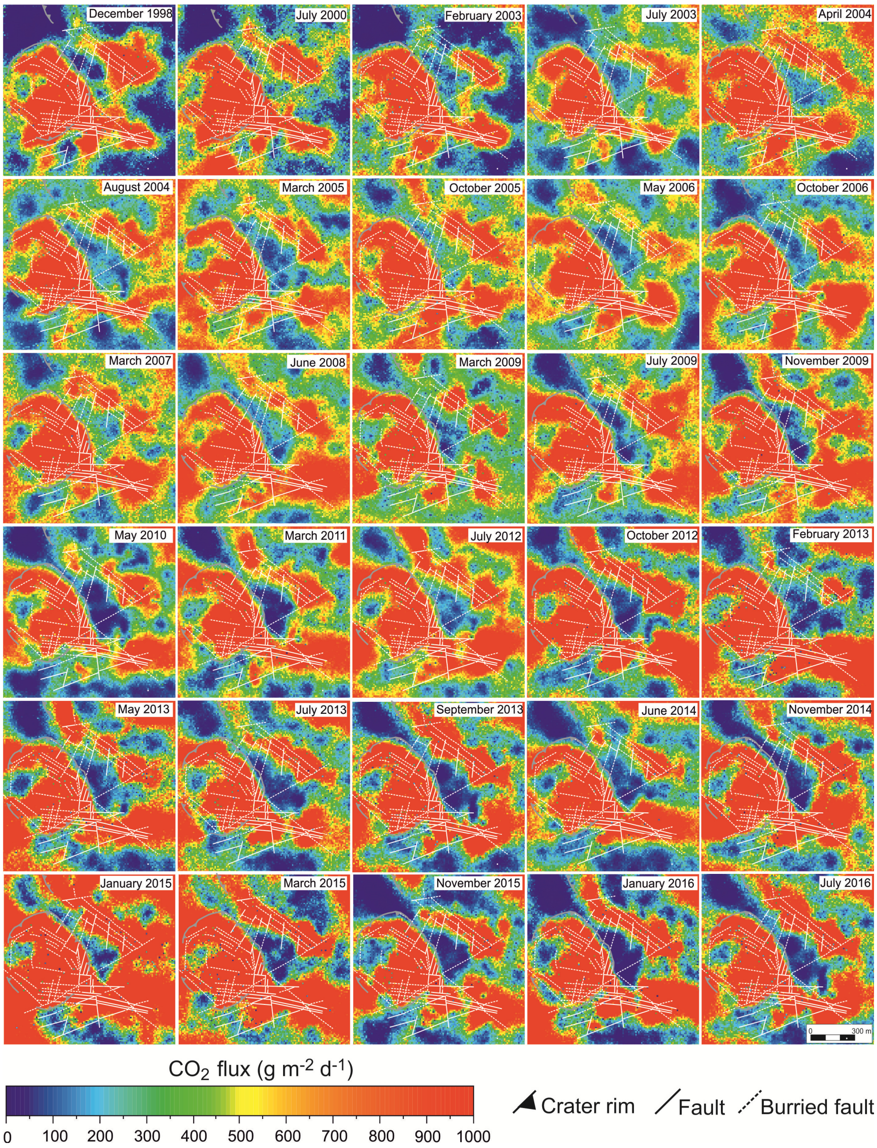

Figure S5. Maps of CO_2 flux of Solfatara in the 1998-2016 period. The maps were realized with the software Surfer, Version 11.0.642 (http://www.goldensoftware.com/products/surfer)

Supplementary Table S1
Parameters of the variogram models showed in Supplementary Information Fig. S2 and used to produce the CO_2 flux maps of Solfatara with the Sequential Gaussian Simulation method,

Data	Variogram model	nugget	sill	range (m)
01/12/1998	spherical	0.17	1.00	340
01/07/2000	spherical	0.26	1.00	330
18/02/2003	spherical	0.24	1.00	290
01/07/2003	spherical	0.35	1.00	270
09/04/2004	spherical	0.37	1.00	290
30/08/2004	spherical	0.20	1.00	285
24/03/2005	spherical	0.32	1.10	220
17/10/2005	spherical	0.35	1.00	270
27/05/2006	spherical	0.30	1.00	310
30/10/2006	spherical	0.27	1.00	280
01/03/2007	spherical	0.35	1.07	270
27/06/2008	spherical	0.33	1.11	290
18/03/2009	spherical	0.35	1.15	270
07/07/2009	spherical	0.25	1.13	300
24/11/2009	spherical	0.20	1.10	245
17/05/2010	spherical	0.16	1.18	310
10/03/2011	spherical	0.22	1.12	280
18/06/2012	spherical	0.25	1.00	240
01/10/2012	spherical	0.26	1.26	285
28/02/2013	spherical	0.32	1.10	300
20/05/2013	spherical	0.32	1.10	265
02/07/2013	spherical	0.30	1.10	275
25/09/2013	spherical	0.27	1.12	300
06/06/2014	spherical	0.25	1.10	325
22/09/2014	spherical	0.25	1.00	230
26/01/2015	spherical	0.26	1.06	240
20/03/2015	spherical	0.30	1.08	270
06/11/2015	spherical	0.20	1.05	290
20/01/2016	spherical	0.20	1.15	280
06/06/2016	spherical	0.22	1.11	290

Supplementary Dataset

Supplementary Dataset S1: CO_2 fluxes from soil (g m^{-2} d^{-1}) and soil temperatures (°C) measured at Solfatara of Pozzuoli in 30 surveys performed from 1998 to 2016.
